# Unexpected *Brucella suis* Biovar 2 Infection in a Dairy Cow, Belgium

**DOI:** 10.3201/eid1912.130506

**Published:** 2013-12

**Authors:** David Fretin, Marcella Mori, Guy Czaplicki, Christian Quinet, Benoît Maquet, Jacques Godfroid, Claude Saegerman

**Affiliations:** Veterinary and Agro-chemical Research Centre, Brussels, Belgium (D. Fretin, M. Mori);; Association Régionale de Santé et d’Identification Animales, Loncin, Belgium (G. Czaplicki, C. Quinet);; Veterinary Practice, Corenne, Belgium (B. Maquet);; Norwegian School of Veterinary Science, Tromsø, Norway (J. Godfroid);; University of Liège Faculty of Veterinary Medicine, Liège, Belgium (C. Saegerman)

**Keywords:** Brucella suis biovar 2, Brucella suis, brucellosis, cattle, wild boar, hunting, biosecurity, cow, Belgium, bacteria, bovine

**To the Editor:** Belgium was declared free of bovine brucellosis by the European Union in 2003 (http://eur-lex.europa.eu/LexUriServ/LexUriServ.do?uri=OJ:L:2003:156:0074:0078:EN:PDF). To maintain this status, the Federal Agency for the Safety of the Food Chain implemented a monitoring program, approved by the European Union, that consists of random serologic surveys and mandatory reporting of spontaneous abortion. This reporting enabled the detection of 2 outbreaks of bovine brucellosis in cattle caused by *Brucella abortus* biovar 3, in 2010 and 2012, but the origin of these outbreaks has not been identified.

As part of an epidemiologic survey conducted to prevent the spread of the infection, ELISA testing (Brucellosis Antibody Test Kit; Idexx, Hoofddorp, the Netherlands) was performed on bulk milk samples from 9,013 dairy farms in the country; 75 farms had positive test results and were classified as reactor farms. All cows in milk production on these farms were serologically tested, first by using slow agglutination test with the addition of EDTA to the antigen, and then, if results were positive, by a commercial ELISA. If results of the ELISA were positive, a confirmatory internal ELISA was performed at the national reference laboratory. A total of 41 seropositive cows from 27 farms were identified. All confirmed seropositive cows were slaughtered for bacteriologic investigation; all had negative test results for *B. abortus*.

On March 23, 2012, bulk milk sample testing for a farm in the province of Namur showed positive results. Testing performed in January 2011 on milk collected from the same farm had yielded negative results. The 150 cattle (including 55 dairy cows) on this farm were further serologically tested. One nonpregnant dairy cow had positive test results by slow agglutination test and ELISAs and was slaughtered on April 23, 2012. The cow was >4 years old, born in the farm, last calved in March 2011, and showed no clinical sign of brucellosis.

Bacteriologic examination was conducted on spleen, uterus, lymph nodes, and udder tissue samples; *Brucella* spp. were cultured from the spleen and uterus. Bacterial colonies grew on *Brucella* agar supplemented with 5% horse serum in the presence of basic thionine and safranine O; CO_2_ was not required for growth, and H_2_S was not produced. The isolates showed catalase, oxidase, and urease activity, a biochemical profile typical of *B. suis* biovar 2; identity was confirmed by real-time PCR on DNA extracted directly from the uterus (*1*). 

A stamping out with compensation policy was implemented for this farm by the Federal Agency for the Safety of the Food Chain, according to European Union regulations, and subsequent epidemiologic investigations were performed. The farm owner is not a hunter. The culture-positive cow originated from a group of 10 nonpregnant or dry dairy cows that had been held in the same pasture, distant from the main farm structures, during October 15–November 15, 2011; during the stamping out process, a second dairy cow from this group had a positive test result by ELISA.

Hunting of wild boar (*Sus scrofa*) had been organized during September–December 2011 in the adjacent forest, and wild boar offal was discarded in a corner of the pasture, with no biosecurity precautions. A recent study confirmed the high prevalence of *B. suis* biovar 2 infection in wild boars in this province (*2*). These findings suggest that these animals were naturally infected with *B. suis* biovar 2; because of the period between infection and testing, the results indicate that antibodies can be detected in cattle by ELISA performed on milk or serum >16 weeks after infection.

Blood samples were taken from the farmer, his wife, and their 2 children, all of whom regularly consumed raw milk. No clinical signs or symptoms suggestive of brucellosis were reported, and slow agglutination test results for all family members were negative (titer <160), which suggests they had no exposure to *B. suis* biovar 2 (*3*). A total of 111 cattle carcasses, including that of the second seropositive cow, were sampled at the abattoir, and all other samples were negative for *Brucella* spp.

Our findings indicate that preventive measures against the spread of pathogens such as *Brucella* spp. must be implemented by hunters (i.e., awareness campaigns, biosecurity education, and responsible hunting practices). In addition, biochemical typing of *Brucella* spp. is necessary to trace the source of infections (*4**,**5*), and epidemiologic inquiry of positive test result(s) should be conducted to identify or exclude bovine brucellosis and to investigate possible *B. suis* biovar 2 infections. Our bacteriologic results (absence of isolation of *B. suis* biovar 2 from all samples collected at the abattoir) suggest that stamping out is not necessary because *B. suis* biovar 2 is not likely to be transmitted between cattle because they are spillover hosts, not preferential hosts for *B. suis* biovar 2, and are thus not likely to sustain the infection. Finally, from a veterinary public health perspective, *B. suis* biovar 2 has a low residual pathogenicity in humans (*5**,**6*).

## References

[1] Fretin D, Whatmore AM, Al Dahouk S, Neubauer H, Garin-Bastuji B, Albert D, *Brucella suis* identification and biovar typing by real-time PCR. Vet Microbiol. 2008;131:376–85 . 10.1016/j.vetmic.2008.04.003 18499359

[2] Grégoire F, Mousset B, Hanrez D, Michaux C, Walravens K, Linden A. A serological and bacteriological survey of brucellosis in wild boar (*Sus scrofa*) in Belgium. BMC Vet Res. 2012;8:80. 10.1186/1746-6148-8-80 22709889 PMC3503824

[3] Corbel MJ. Brucellosis in humans and animals. World Health Organization in collaboration with the Food and Agriculture Organization of the United Nations and World Organisation for Animal Health. Geneva: World Health Organization; 2006.

[4] Saegerman C, Berkvens D, Godfroid J, Walravens K. Bovine brucellosis. In: Blancou P, Chermette R, editors. Infectious and parasitic disease of livestock. Paris: Lavoisier and Commonwealth Agricultural Bureau International; 2001. p. 991–1021.

[5] Godfroid J, Nielsen K, Saegerman C. Diagnosis of brucellosis in livestock and wildlife. Croat Med J. 2010;51:296–305 . 10.3325/cmj.2010.51.296 20718082 PMC2931434

[6] Alton GG. *Brucella suis.* In: Nielsen K, Duncan JR, ed. Animal brucellosis. Boca Raton (FL): CRC Press; 1990. p. 411–422.

